# Targeting Autophagy, Apoptosis, and Oxidative Perturbations with Dapagliflozin Mitigates Cadmium-Induced Cognitive Dysfunction in Rats

**DOI:** 10.3390/biomedicines11113000

**Published:** 2023-11-08

**Authors:** Hany H. Arab, Ahmed H. Eid, Shuruq E. Alsufyani, Ahmed M. Ashour, Azza A. K. El-Sheikh, Hany W. Darwish, Fatma M. Sabry

**Affiliations:** 1 Department of Pharmacology and Toxicology, College of Pharmacy, Taif University, P.O. Box 11099, Taif 21944, Saudi Arabia; s.alsofyani@tu.edu.sa; 2 Department of Biochemistry, Faculty of Pharmacy, Cairo University, Cairo 11562, Egypt; 3 Department of Pharmacology, Egyptian Drug Authority (EDA)—Formerly NODCAR, Giza 12654, Egypt; drahmedhamdy2007@yahoo.com (A.H.E.); fatmasabry@ymail.com (F.M.S.); 4 Department of Pharmacology and Toxicology, College of Pharmacy, Umm Al Qura University, P.O. Box 13578, Makkah 21955, Saudi Arabia; amashour@uqu.edu.sa; 5 Basic Health Sciences Department, College of Medicine, Princess Nourah bint Abdulrahman University, P.O. Box 84428, Riyadh 11671, Saudi Arabia; aaelsheikh@pnu.edu.sa; 6 Department of Pharmaceutical Chemistry, College of Pharmacy, King Saud University, P.O. Box 11451, Riyadh 11451, Saudi Arabia; hdarwish@ksu.edu.sa

**Keywords:** dapagliflozin, cadmium, neurotoxicity, autophagy, apoptosis, oxidative stress

## Abstract

Cognitive decline and Alzheimer-like neuropathology are common manifestations of cadmium toxicity. Thanks to its antioxidant/anti-apoptotic features, dapagliflozin has demonstrated promising neuroprotective actions. However, its effect on cadmium-induced neurotoxicity is lacking. The present work aimed to examine whether dapagliflozin could protect rats from cadmium-evoked cognitive decline. In this study, the behavioral disturbances and hippocampal biomolecular alterations were studied after receiving dapagliflozin. Herein, cadmium-induced memory/learning decline was rescued in the Morris water maze, novel object recognition task, and Y-shaped maze by dapagliflozin. Meanwhile, the hippocampal histopathological abnormalities were mitigated. The molecular mechanisms revealed that dapagliflozin lowered hippocampal expression of p-tau and Aβ42 neurotoxic proteins while augmenting acetylcholine. The cognitive enhancement was triggered by hippocampal autophagy stimulation, as indicated by decreased SQSTM-1/p62 and Beclin 1 upregulation. Meanwhile, a decrease in p-mTOR/total mTOR and an increase in p-AMPK/total AMPK ratio were observed in response to dapagliflozin, reflecting AMPK/mTOR cascade stimulation. Dapagliflozin, on the other hand, dampened the pro-apoptotic processes in the hippocampus by downregulating Bax, upregulating Bcl-2, and inactivating GSK-3β. The hippocampal oxidative insult was mitigated by dapagliflozin as seen by lipid peroxide lowering, antioxidants augmentation, and SIRT1/Nrf2/HO-1 pathway activation. In conclusion, dapagliflozin’s promising neuroprotection was triggered by its pro-autophagic, anti-apoptotic, and antioxidant properties.

## 1. Introduction

Exposure to cadmium has been assumed unavoidable due to its long biological half-life in humans and its ubiquitous usage in agricultural and industrial activities [1,2]. Repeated cadmium exposure prompts severe neurotoxicity, including memory loss and Alzheimer’s disease (AD)-like symptoms. In this context, various epidemiological investigations have confirmed that prolonged exposure to cadmium metal in humans results in reduced cognitive performance [2,3]. As a result of cadmium-induced neurotoxicity, cognitive impairment, memory loss, and learning difficulties are common symptoms [4,5].

The pathophysiology of cadmium neurotoxicity revealed that the progression of cognitive deficits is tightly linked to neuronal oxidative stress [1]. Combined with the depletion of neuronal antioxidants, cadmium induces excessive amounts of reactive oxygen species (ROS). Moreover, cadmium neurotoxicity also implicates disturbance in the cascade of the nuclear factor erythroid 2-related factor-2 (Nrf2)/heme oxygenase-1 (HO-1). In perspective, it has been shown that cadmium may inhibit or stimulate the Nrf2/HO-1 pathway in vivo, either inactivating [4] or stimulating [6] this redox-associated pathway. Accordingly, additional exploration is needed to determine the specific role of Nrf2/HO-1 in the neuropathology of cadmium-triggered hippocampal damage in vivo. In the same regard, murine models of cognitive dysfunction demonstrated that cadmium triggers downregulation of silent information-regulated transcription factor 1 (SIRT1) expression [5,7]. Accumulation of the oxidative events and cell stressors instigates neuronal apoptotic cell death driven majorly by the mitochondrial pathway. This is associated with overshooting of pro-apoptotic signals, such as B cell lymphoma-2 (Bcl-2)-associated x protein (Bax) alongside decreased anti-apoptotic cues [5,6,8], resulting in hippocampal neuronal loss and consequent learning and memory deficits in animals [5].

In the neuropathology of cadmium-induced cognitive deficits, autophagy has recently been characterized as an important factor. Basically, autophagy favors cellular survival by ridding the cells of noxious signals including misfolded proteins and damaged mitochondria [8]. With respect to cadmium-triggered neurotoxicity, the in vitro investigations have revealed conflicting findings in neuronal cells where stimulation [9], as well as inactivation [10,11,12] of autophagy, have been described. Furthermore, the in vivo exploration of autophagy in experimental animals has been insufficiently addressed [6]. Thus, the present work focuses on the role of autophagy in mediating cadmium-triggered neurotoxicity in vivo in rats.

The association between cadmium exposure and AD-like manifestations has lately been revealed. In perspective, the preclinical studies in murine models have demonstrated defective memory and learning ability upon repeated exposure to cadmium [4,5]. These cognitive deficits have been associated with the precipitation of amyloid plaques in the brain hippocampus of rodents. Likewise, a murine model of cadmium-induced cognitive impairment has shown that hippocampal phospho-tau (p-tau) is deposited in the form of neurofibrillary tangles [3].

**Figure 1:**
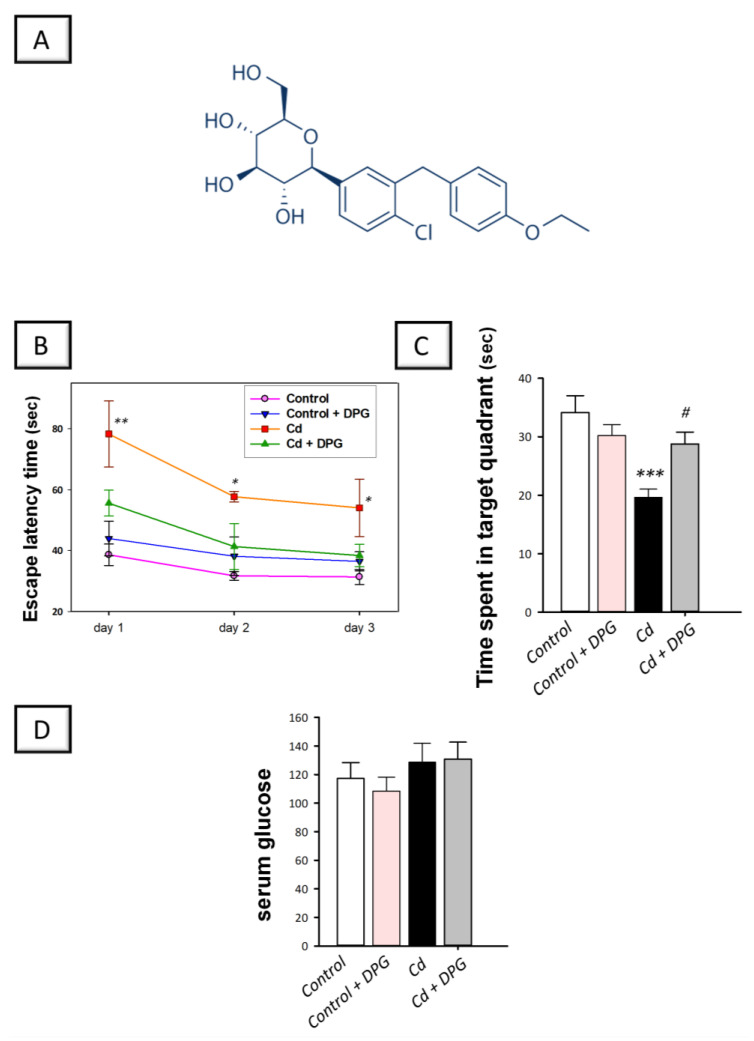
Dapagliflozin improved memory retention in cadmium-intoxicated rats. (**A**) Illustration of dapagliflozin’s chemical structure. During the Morris water maze (MWM) test, three days of training (4 times per day) were conducted in which the concealed platform was positioned in the southeastern quadrant. A probe test was conducted twenty-four hours later, during which the hidden platform was removed. (**B**) Dapagliflozin tended to lower the time it took to locate the concealed platform during the training sessions; however, the change did not reach statistical significance. (**C**) In the probe test, the time spent within the targeted quadrant was statistically significantly increased by dapagliflozin, indicating improved retention memory in animals. (**D**) Glucose levels in serum of animals. The mean ± standard error (SE) was presented in the graph (*n* = 6). Statistical comparisons versus the control group were demonstrated by *** *p* < 0.001, ** *p* < 0.01, or * *p* < 0.05. Statistical comparisons versus the Cd group were demonstrated by ^#^ *p* < 0.05 (Bonferroni’s test and one-way ANOVA). Cd, cadmium chloride (5 mg/kg/day, via gavage for 2 months); DPG, dapagliflozin (1 mg/kg/day, by gavage for 2 months).

As a selective and potent sodium-glucose co-transporter 2 (SGLT2) inhibitor, dapagliflozin (DPG) is used in the clinical setting to lower hyperglycemia in type 2 diabetic patients (the chemical structure is shown in Figure 1A) [13]. In the clinical setting, it has demonstrated a low risk of hypoglycemia. Consistently, DPG does not lower blood glucose levels in normoglycemic rodents, as established in several experimental models [14]. Virtually, significant expression of SGLT2 has been reported in the mammalian central nervous system (CNS) as characterized in the hippocampus, cerebellum, and blood–brain barrier (BBB) endothelial cells [15]. Moreover, the penetration of SGLT2 inhibitors to the CNS has been pointed out where these lipid-soluble drugs can cross the BBB, particularly, during inflammatory neurological disorders [15,16]. In the context of its neuroprotective potential, DPG has shown marked neuroprotection in several experimental models as evidenced by the improvement of the cognitive deficit in 3-nitropropionic acid neurotoxicity [17] and high-fat diet-evoked obesity [18]. Evidence also shows that DPG ameliorates behavioral disruption associated with chronic stress-triggered depression-like behavior [19] and rotenone-induced Parkinson’s disease manifestations [16]. Even with these promising neuroprotective properties, the possibility of DPG to counteract cadmium-triggered learning and memory impairment in rats has not been investigated. Thus, the aim of the present study is to explore whether the reported antioxidant [14,16,20] and anti-apoptotic features [16,17,20] of DPG, may lower the neuronal damage and cognitive decline associated with cadmium intoxication in rats. In perspective, the behavioral changes linked to cognitive decline were examined together with the molecular manifestations in the hippocampus, the main brain region in charge of orchestrating learning and memory activities [4,5,6]. Mechanistically, the current study focused on hippocampal alterations of the redox milieu, apoptosis, and autophagy. In the present study protocol, DPG’s neuroprotective potential was examined away from its glucose-lowering competence by using normoglycemic rats [14]. This approach is based on the fact that hyperglycemia itself may trigger neuronal damage [21].

## 2. Materials and Methods

### 2.1. Chemicals

AstraZeneca Pharmaceutical Company provided dapagliflozin (AstraZeneca, Cairo, Egypt). Cadmium chloride was acquired from a commercial supplier (Sigma-Aldrich Co., St. Louis, MO, USA; Cat. # 202908). The sources for reagents/kits are specified for each assay. The fine chemicals and other reagents were of the highest analytical grade.

### 2.2. Experimental Animals

In the current methodology, the animal protocol and handling were applied in accordance with the Laboratory Animals Guide for Care and Use (US-NIH, USA, Publication No. 85-23, revised 1996). Ethics approval was obtained from the Research Ethics Committee of the Egyptian Drug Authority (Approval ID: NODCAR/I/9/2022).

For the current study investigation, 8 to 10-week-old Wistar albino rats were used. Animals were procured from the Breeding Unit of the Egyptian Drug Authority, Giza, Egypt, and animals’ weights ranged between 160 to 200 g. At the animal facility, animals were kept in polycarbonate cages under fixed experimental conditions, including a 12 h dark/light cycle, temperature of 21–24 °C, and humidity of 40%. Rats had a two-week acclimatization period with unlimited access to drinking water and laboratory chow.

### 2.3. Preclinical Animal Model

To lessen suffering and stress, animals were handled with care. The experimental study was carried out using four experimental groups (each group included 10 rats). To this end, a blinded technician randomly separated animals into four experimental groups, as characterized in Table 1.

### 2.4. Morris Water Maze (MWM)

Assessment of learning ability and spatial memory was examined by Morris test protocol [29]. Essentially, the maze consists of a circular tank (0.6 m high × 1.5 m in diameter) containing water at 25 °C and divided into four quadrants. A platform was submerged under the water surface within the target quadrant (10 cm diameter). For each rat, three consecutive days of training (memory acquisition trials) were conducted four times per day; each trial lasted one minute. During each acquisition trial, the rats were allowed to find the hidden platform within the target area. On the fourth day (retrieval trial or probe-test session), the rat was free to explore the tank for 1 min with the concealed platform. The period spent by each rat within the targeted quadrant represents a measure of memory retention.

### 2.5. Novel Object Recognition Test (NORT)

Rodents are evaluated on their ability to recognize a novel object in the environment by completing the NOR task (NORT) using a box (80 × 80 × 40 cm): white in color with black grid lines [30]. Basically, NORT examines rodents’ natural preferences for novel objects. Three phases comprise the task procedure: habituation, familiarization, and testing. In the habituation phase, the animals are free to roam the open-field arena without objects for 10 min. During the familiarization (training) phase, the rat was placed for five minutes in the open-field arena for the exploration of two identical objects (A + A′). When a rat touches an object or approaches it with its nose at a distance of less than two centimeters, it is exploring it. To eliminate the possibility of bias caused by odor cues, the box and objects were cleaned with 70% ethanol after each session. Herein, we evaluated the long-term memory in animals, as the testing phase was applied 24 h after training. The test procedures were conducted by returning the animal to the open-field arena to explore one familiar old object (A) in addition to another novel object (B). The sniffing (exploration) times were measured for the old (A) and novel (B) objects, and in the results, we presented the mean object exploration time (seconds; for the old and novel objects) alongside the total exploration time (seconds). Herein, animals with good recognition memory are more likely to explore the novel object (B) in comparison to the old object (A). Then, we calculated the discrimination ratio by dividing the time spent exploring object (B) divided by the total exploration time spent on object (A) + object (B).

### 2.6. Y-Shaped Maze

We evaluated the short-term memory in animals using the Y-maze apparatus with 3 equal arms (each, L: 40 cm, H: 15 cm, and W: 10 cm) [31]. In the training procedure, the rat was placed in the center of the maze, with full access to move through two open arms and one closed arm for 10 min [29]. A thorough cleaning of the arms was performed after each rat had been removed in order to eliminate any residual odors. The test was applied 1 h post-training by allowing the animal to explore the maze with the 3 arms open. For each rat, we recorded the time spent exploring the new (unexplored) arm in comparison with the old arm. In this regard, animals with good spatial memory are more likely to enter a new (unexplored) arm. Then, a ratio was calculated between the time spent in the new arm and the time spent in the old arm.

### 2.7. Collecting and Processing Brain Tissue

After performing behavioral tests, animals were euthanized under anesthesia immediately after collecting blood by cardiac puncture. There were then two subsets of each group. First, four randomly selected brains were quickly isolated and preserved in 10% formalin for immunohistochemistry and histopathology. For biochemical investigations, the hippocampi from the second subset (*n* = 6) were rapidly removed, and stored at −80 °C.

### 2.8. Measurement of Serum Glucose

As recommended by the provider, serum samples (HUMAN Diagnostics, Wiesbaden, Germany; Cat. # BD184) were tested for glucose using HUMAN colorimetric assay kits. In order to determine the final color, the wavelength was set at 500 nm.

### 2.9. Histopathology

A hematoxylin and eosin (H&E) staining was conducted to determine neuronal damage in hippocampal tissues. The procedures were conducted by blinded scientists to eliminate bias [32]. The samples were embedded in Paraplast tissue media after being fixed in neutral buffered formalin (10%). A series of coronal sections of the brain was used in order to demonstrate hippocampal regions. Four-micrometer sections were cut by a rotary microtome and mounted on a glass slide. A light microscopic examination (Leica Microsystems, Wetzlar, Germany) was performed by staining the tissue with H-E. In this regard, six fields were photographed in a non-overlapping manner [33,34].

Quantification of histological damage, including microglial infiltration and pyknosis, was based on a scoring system that ranged from 0 to 4 [35,36]. As per the scoring system, 4 represents the lesion in >60% area, 3 indicates the lesion in 40–60% area, 2 indicates the lesion in 10–40% area, 1 indicates the lesion in >10% area, and 0 represents the absence of lesions.

### 2.10. Immunohistochemistry

For Bcl-2 and Bax immunohistochemistry, sections of brain tissues were processed as instructed by the manufacturer as previously described [37,38]. After deparaffinization and antigen retrieval, treatment with 0.3% hydrogen peroxide was applied to block tissue peroxidase activity. Incubation of the sections with the primary antibodies was conducted overnight at 4 degrees Celsius with either anti-Bax (Cat. # 33-6600; dilution of 1:100) or anti-Bcl-2 (Cat. # PA1-30411; dilution of 1:100, Thermo Fisher Scientific, Rockford, IL, USA). The tissue sections were washed with PBS prior to incubation with the HRP-tagged secondary antibody Envision kit (DAKO). In order to visualize the immune reaction, the sections were incubated with diaminobenzidine (DAB) chromogen and examined microscopically. Area percentages of immunohistochemistry were assessed by scanning six non-overlapping fields within the hippocampal CA3 region at random. Immunohistochemistry data was processed using the Leica software version 3.8 (Leica Microsystems, Wetzlar, Germany).

### 2.11. Evaluation of Acetylcholine Esterase, Acetylcholine, and Caspase-3 Activity

A commercial vendor provided the fluorescent activity kit used to quantify the activity of acetylcholinesterase (Cat. # KT-708, Kamiya, Seattle, WA, USA) whereas Elabscience (Wuhan, China) supplied the ELISA kit used to measure Aβ42 (Cat. # E-EL-R1402). In this experiment, the excitation wavelength was 370 nm while the fluorescent emission was measured at 510 nm. Moreover, Cloud-Clone Corporation (Houston, TX, USA) supplied the ELISA kit used to measure acetylcholine levels (Cat. # CEA912Ge) whereas Sigma-Aldrich (St. Louis, MO, USA) provided the colorimetric assay kit for hippocampal caspase-3 activity (Cat. # CASP-3-C). The final color’s optical density was quantified at 405 nm.

### 2.12. Evaluation of Hippocampal p-tau, Aβ42, and p-GSK-3β

For the neurotoxic signals, Fine Biotech Company (Wuhan, China) provided the ELISA kit used to measure p-tau levels (Cat. # ER1304) whereas Elabscience (Wuhan, China) supplied the ELISA kit to measure Aβ42 (Cat. # E-EL-R1402). For both kits, a microplate reader was used to measure the absorbance at 450 nm. Moreover, Cell Signaling Technology (Danvers, MA, USA) provided the specific kits for the assay of p-GSK-3β(Ser9) (Cat. # 7311C) and total GSK-3β (Cat. # 7265C), as described by the manufacturer. A microplate reader was used to measure the absorbance at 450 nm.

### 2.13. Determination of Autophagy Events

For the autophagy markers, SunLong Biotech. (Hangzhou, Zhejiang, China) provided the ELISA kit used to measure SQSTM-1/p62 (Cat. # SL1363Ra). Likewise, AFG Bioscience (Northbrook, IL, USA) provided the specific kit for the assay of Beclin 1 (Cat. # EK720982). A microplate reader was used to measure the absorbance at 450 nm. For AMPK/mTOR pathway, an ELISA kit manufactured by RayBiotech (Norcross, GA, USA) was employed to assay the protein levels of total AMPK and p-AMPK(Ser487) (Cat. # PEL-AMPKA-S487-T). Moreover, Cell Signaling Technology (Danvers, MA, USA) provided specific kits for the assay of p-mTOR (Cat. # 7976C) and total mTOR (Cat. # 7974C), as specified by the manufacturer. A microplate reader was used to measure the absorbance at 450 nm. In accordance with vendors’ instructions and previous literature [39], fold changes were expressed for the values of p-mTOR/total mTOR and p-AMPK/total AMPK.

### 2.14. Evaluation of the Redox Milieu

The thiobarbituric acid-reactive substance assay was used to measure lipid peroxide levels (measured as malondialdehyde) [37]. The final color of the product was detected at a wavelength of 535 nm. According to the instructions provided by the manufacturer, we used a specific glutathione peroxidase kit for testing its activity (Sigma-Aldrich, St. Louis, MO, USA). Through the use of kinetic software, we monitored the decrease in absorbance at 340 nm. To assess total protein content, the assay of Lowry et al. [32] was applied. AFG Bioscience (Northbrook, IL, USA) provided the ELISA kit used to measure SIRT1 (Cat. # EK720561). The assay of Nrf2 protein expression was conducted in the nuclear compartment. A specific extraction kit for the nuclear content (Cayman Chemical, Ann Arbor, MA, USA) was used to isolate total nuclear proteins, according to the instructions of the manufacturer (Cat. # 10009277). Moreover, an ELISA kit manufactured by AFG Bioscience (Northbrook, IL, USA) was used to assay the protein levels of Nrf2 (Cat. # EK720003). An ELISA kit from Elabscience (Wuhan, China) was used to determine the protein levels of HO-1 (Cat. # E-EL-R0488). A microplate reader was used to measure the absorbance at 450 nm.

### 2.15. Data Presentation and Statistical Analysis

Graph Pad Prism version 9 software was used to conduct all statistical analyses (San Diego, CA, USA), and *p* < 0.05 was used as the minimum for statistical significance. To represent data, we used the mean + standard error (SE). Data normal distribution was examined by Shapiro–Wilk test. For the purpose of estimating the statistically significant differences among experimental groups, Bonferroni’s test and one-way analysis of variance (ANOVA) test were implemented for parametric values. Likewise, for non-parametric values, Dunn’s test and Kruskal–Wallis test were used.

## 3. Results

### 3.1. Dapagliflozin Improved Memory Retention in Cadmium-Intoxicated Rats

Rats were tested on their spatial memory by the Morris water maze (MWM) to explore whether dapagliflozin (DPG) can alleviate the cadmium-evoked cognitive deficits in rats [29]. In the hidden platform training sessions (Figure 1B), cadmium-intoxicated rats suffered defective spatial learning ability, as revealed by a statistically significantly longer escape latency time on days 1 (*p* < 0.01), 2 (*p* < 0.05), and 3 (*p* < 0.05) of training by 102.8%, 49.5%, and 40%, respectively, to locate the hidden platform in comparison to control rats (day 1: F (3, 20) = 6.937, *p* = 0.0027; day 2: F (3, 20) = 3.286, *p* = 0.0408; day 3: F (3, 20) = 3.697, *p* = 0.0378). Administration of DPG to cadmium-intoxicated rats tended to lower the escape latency time to identify the hidden platform; however, the change did not reach statistical significance. In the probe test (Figure 1C), cadmium-intoxicated animals demonstrated a statistically significant (*p* < 0.001) decline in the time spent within the targeted quadrant by 42.4% where the hidden platform was located, reflecting defective retention memory, in comparison to control rats (F (3, 20) = 8.284, *p* = 0.0009). A statistically significant (*p* < 0.05) increase in the time spent within the targeted quadrant by 46.2% was observed after DPG was administered to cadmium-intoxicated rats, implying improvement of the retention memory. Regarding the levels of serum glucose (Figure 1D), all experimental groups did not exhibit statistically significant differences in this parameter, implying that DPG exerts no hypoglycemia in animals (F (3, 20) = 0.8103, *p* = 0.5030). Together, these findings indicate that DPG can help the recovery of rats from cadmium-induced retention memory dysfunction.

### 3.2. Dapagliflozin Rescued Recognition Memory Deterioration in Cadmium-Intoxicated Rats

**Figure 2:**
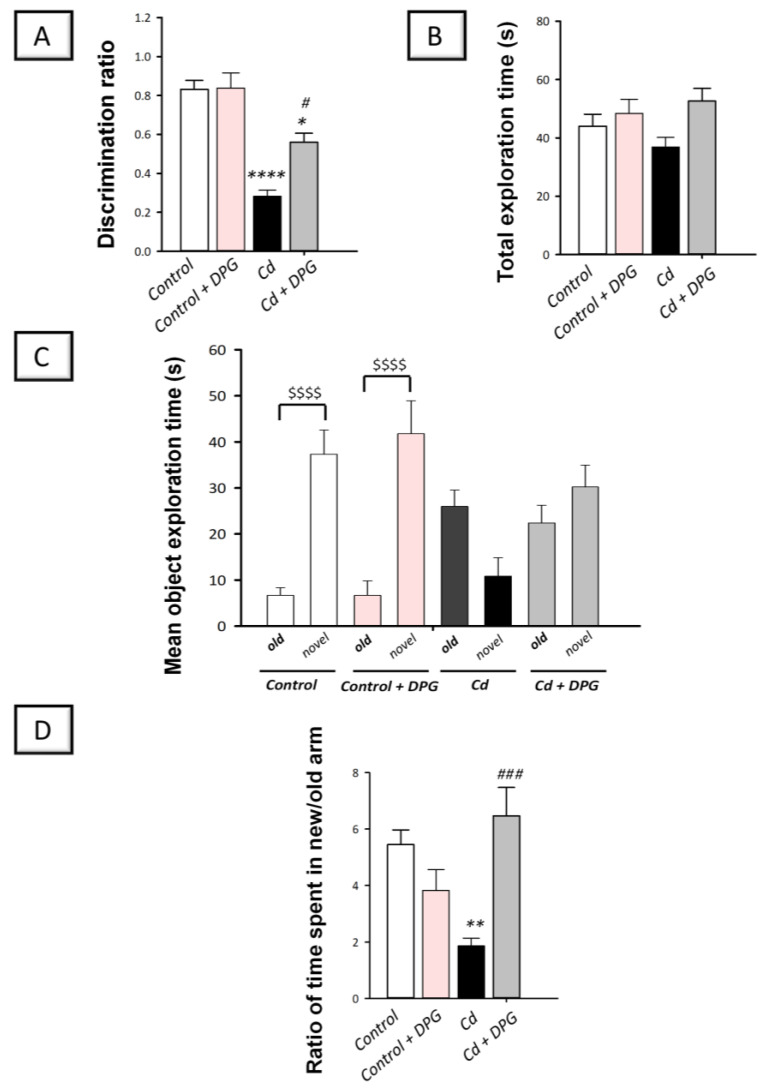
Dapagliflozin rescued the recognition memory dysfunction in cadmium-intoxicated rats. (**A**) In the novel object recognition task (NORT), the discrimination ratio was increased by dapagliflozin, reflecting long-term enhancement in recognition memory. In NORT, we also recorded the total exploration time (**B**) and the mean object exploration time (for the old and novel objects; (**C**)). (**D**) In the Y-maze test, the ratio of the time spent in the new/old arm was elevated by dapagliflozin, affirming short-term improvement in recognition memory. The mean ± standard error (SE) was presented in the graph (*n* = 6). Statistical comparisons versus the control group were demonstrated by **** *p* < 0.0001, ** *p* < 0.01, or * *p* < 0.05. Statistical comparisons versus the Cd group were demonstrated by ^###^ *p* < 0.001, or ^#^ *p* < 0.05. In the mean object exploration time, statistical significance between the old and the novel object exploration time was demonstrated by ^$$$$^ *p* < 0.0001 (Bonferroni’s test and one-way ANOVA). Cd, cadmium chloride (5 mg/kg/day, via gavage for 2 months); DPG, dapagliflozin (1 mg/kg/day, by gavage for 2 months).

Rats were tested on their recognition memory by the novel object recognition task (NORT) and the Y-shaped maze. First, the spontaneous tendency to explore new objects in animals was examined by NORT [30]. Since the test of NORT was conducted 24 h after training, this test examined the long-term ability of DPG to alleviate cadmium-evoked memory deterioration in animals. In Figure 2A, a statistically significant (*p* < 0.0001) reduction in the discrimination ratio was observed in rats intoxicated with cadmium by 66.1%, indicating long-term impairment of the recognition memory (F (3, 20) = 17.38, *p* < 0.0001). This ratio was statistically significantly (*p* < 0.05) increased by 98.6% after DPG was administered to cadmium-intoxicated rats, reflecting recognition memory improvement. Of note, the obtained values for the discrimination ratio in control rats are in agreement with previously published studies [40,41,42,43,44,45]. In addition to the discrimination ratio, we measured the total exploration time (Figure 2B) and the mean object exploration time (for the old and novel objects; Figure 2C). Herein, statistical non-significant changes were detected among the experimental groups regarding the total exploration time (F (3, 20) = 2.594, *p* = 0.0811). On the other hand, the mean exploration time was significantly increased for the novel object versus the old object in the control and DPG-treated control groups (*p* < 0.0001).

Moreover, the potential competence of DPG to alleviate cadmium-evoked memory deterioration in the short term (1 h post-training) was examined using the Y-maze as a valid test for recognition memory [31]. In Figure 2D, a statistically significant (*p* < 0.01) reduction in the ratio of the time spent in the new/old arm was observed in intoxicated rats by 65.8%, indicating a short-term impairment of recognition memory (F (3, 20) = 8.610, *p* = 0.0007). This ratio was statistically significantly (*p* < 0.001) increased by 247.5% after DPG was administered to cadmium-intoxicated rats, indicating recognition memory enhancement. Together, these findings indicate that DPG can help the recovery of rats from cadmium-induced recognition memory deficit.

### 3.3. Dapagliflozin Mitigated Hippocampal Histopathological Signs in Cadmium-Intoxicated Rats

**Figure 3:**
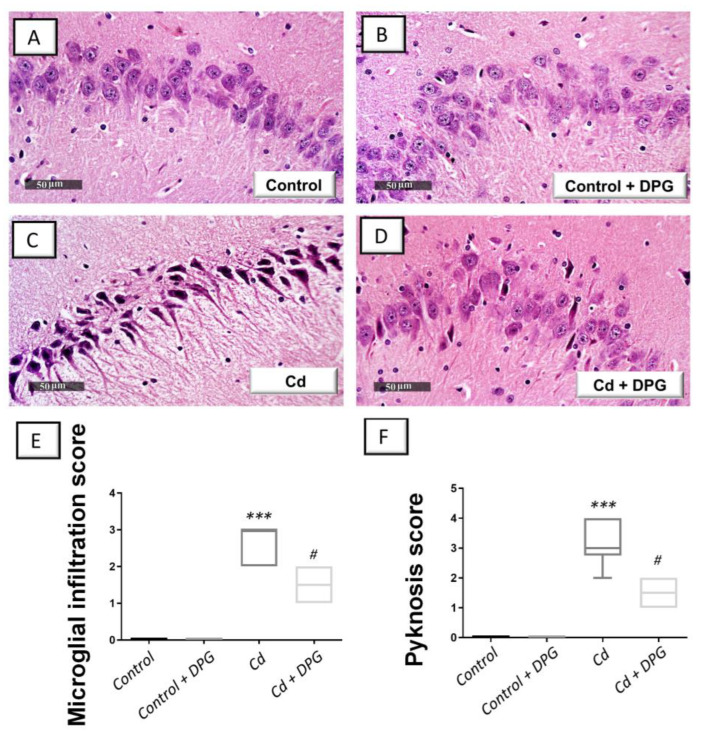
Dapagliflozin lowered the scores of microglial infiltration and pyknosis in the hippocampi of cadmium-intoxicated rats. The histomorphological changes in hippocampal sagittal sections were revealed by light microscopy after hematoxylin and eosin (H-E) staining. The normal architecture of the hippocampus with intact pyramidal neurons was demonstrated in the sections of the control (**A**) and dapagliflozin-treated control group (**B**). (**C**) In contrast, the hippocampus region of the Cd group exhibited marked microglial cell infiltration and pyknosis in pyramidal neurons. (**D**) Lowered records of microglial cell infiltration and pyknosis were observed in Cd + DPG group. (**E**,**F**) Administration of DPG to cadmium-intoxicated animals statistically significantly reduced the scores of microglial infiltration and pyknosis, respectively. The median and interquartile range were presented in the graph (*n* = 6). Statistical comparisons versus the control group were demonstrated by *** *p* < 0.001. Statistical comparisons versus the Cd group were demonstrated by ^#^ *p* < 0.05 (Dunn’s test and Kruskal–Wallis test). Cd, cadmium chloride (5 mg/kg/day, via gavage for 2 months); DPG, dapagliflozin (1 mg/kg/day, by gavage for 2 months).

The potential mitigation competence of DPG of hippocampal histomorphological aberrations evoked by cadmium was explored by light microscopy. The present findings demonstrated that hippocampal sections from the control and control + DPG groups (Figure 3 A,B) revealed normal structure of the hippocampus, intact nuclear and subcellular structures in normal pyramidal neurons, and well-organized layers of hippocampal cells. In contrast, hippocampal sections from Cd group demonstrated marked reactive microglial cell infiltrates and edema in the brain matrix in addition to severe hippocampal degeneration and pyramidal neuron pyknosis (Figure 3C). These pathological findings were mitigated in response to DPG administration to Cd-intoxicated rats. This was manifested by fewer records of microglial cell influx and pyknosis (Figure 3D). These data were corroborated by the scores of microglial cell influx and pyknosis, as depicted in Figure 3E,F. Although cadmium afforded statistically significant elevation (*p* < 0.001) in the scores of microglial infiltration and pyknosis (microglial infiltration scores: H (3, 20) = 19.51, *p* = 0.0002; pyknosis scores: H (3, 20) = 19.75, *p* = 0.0002), these neuropathological scores were statistically significantly (*p* < 0.05) dampened by DPG. These data point to DPG’s competence in attenuating cadmium-evoked histomorphological aberrations and neuronal degeneration.

### 3.4. Dapagliflozin Downregulated Hippocampal Protein Expression of p-tau and Aβ42 While Upregulating the Inactive p-GSK-3β(Ser9) in Cadmium-Intoxicated Rats

**Figure 4:**
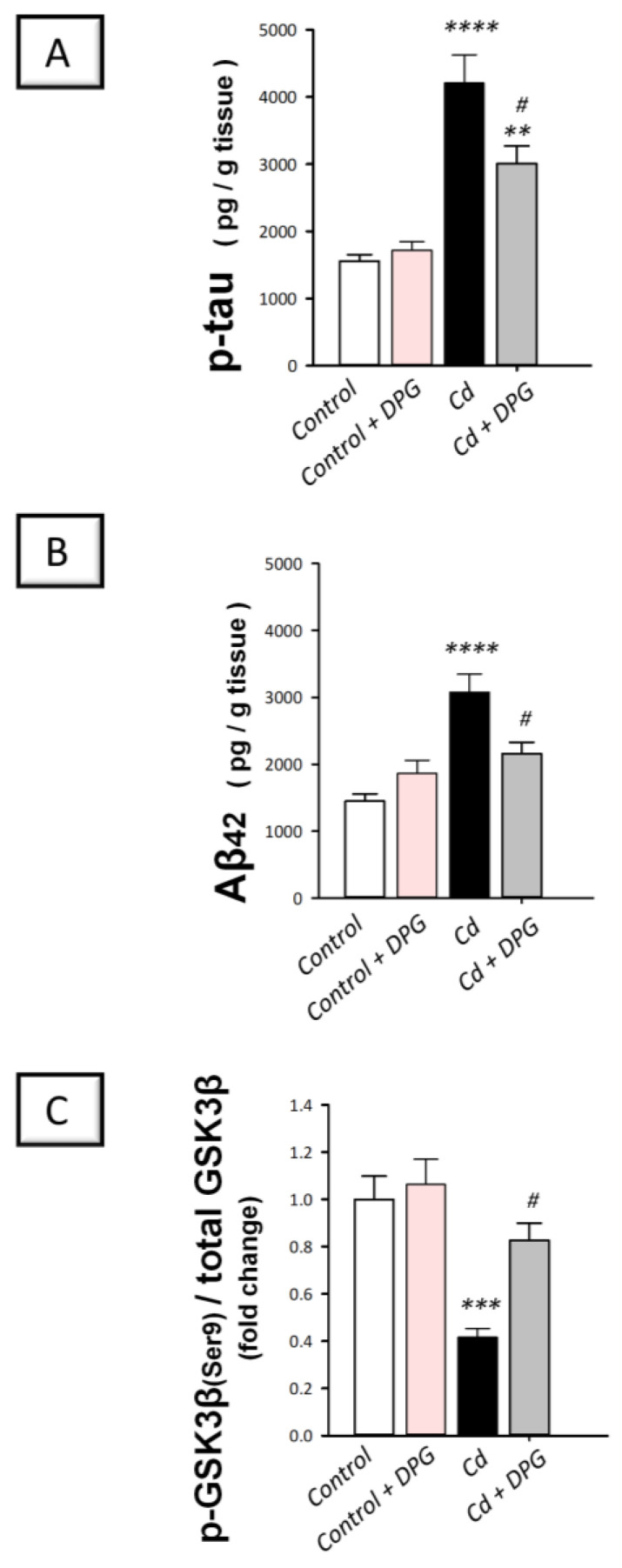
Dapagliflozin decreased hippocampal protein expression of p-tau and Aβ42 while enhancing the expression of the inactive p-GSK-3β (Ser9) in cadmium-intoxicated rats. (**A**) Phosphorylated tau protein expression levels (p-tau). (**B**) The levels of amyloid-beta 42 (Aβ_42_) protein expression. (**C**) Phosphorylated glycogen synthase kinase-3 beta (p-GSK-3β(Ser9)). The mean ± standard error (SE) was presented in the graph (*n* = 6). Statistical comparisons versus the control group were demonstrated by **** *p* < 0.0001, *** *p* < 0.001, or ** *p* < 0.01. Statistical comparisons versus the Cd group were demonstrated by ^#^ *p* < 0.05 (Bonferroni’s test and one-way ANOVA). Cd, cadmium chloride (5 mg/kg/day, via gavage for 2 months); DPG, dapagliflozin (1 mg/kg/day, by gavage for 2 months).

The implicated mechanisms that mediated the cognitive decline in animals were investigated by measuring the neurotoxic signals, such as phosphorylated tau (p-tau) and its upstream effector glycogen synthase kinase-3 beta GSK-3β. Moreover, the protein levels of the noxious amyloid-beta 1-42 (Aβ_42_) were explored. In Figure 4A,B, cadmium instigated a statistically significant (*p* < 0.0001) increase in p-tau, and Aβ_42_ by 170.6% and 112.3%, respectively (p-tau: F (3, 20) = 22.54, *p* < 0.0001; Aβ_42:_ F (3, 20) = 12.59, *p* < 0.0001) together with an obvious activation of the GSK-3β pro-apoptotic kinase, as evidenced by a statistically significant (*p* < 0.001) downregulation in the inactive p-GSK-3β(Ser9) by 58.4%, in comparison to control rats as depicted in Figure 4C (F (3, 20) = 12.34, *p* < 0.0001). Administration of DPG to cadmium-intoxicated rats counteracted these events as seen by the statistically significantly (*p* < 0.05) downregulated expression of p-tau and Aβ_42_ by 28.5% and 29.8%, respectively, while statistically significantly (*p* < 0.05) increasing p-GSK-3β(Ser9) protein expression by 98.7%. Therefore, these findings show that DPG can dampen neurotoxic signals in rats’ hippocampi by downregulating p-tau and Aβ_42_ expression and inactivating GSK-3β.

### 3.5. Dapagliflozin Attenuated Hippocampal Acetylcholine Esterase While Enhancing Acetylcholine Levels in Cadmium-Intoxicated Rats

**Figure 5:**
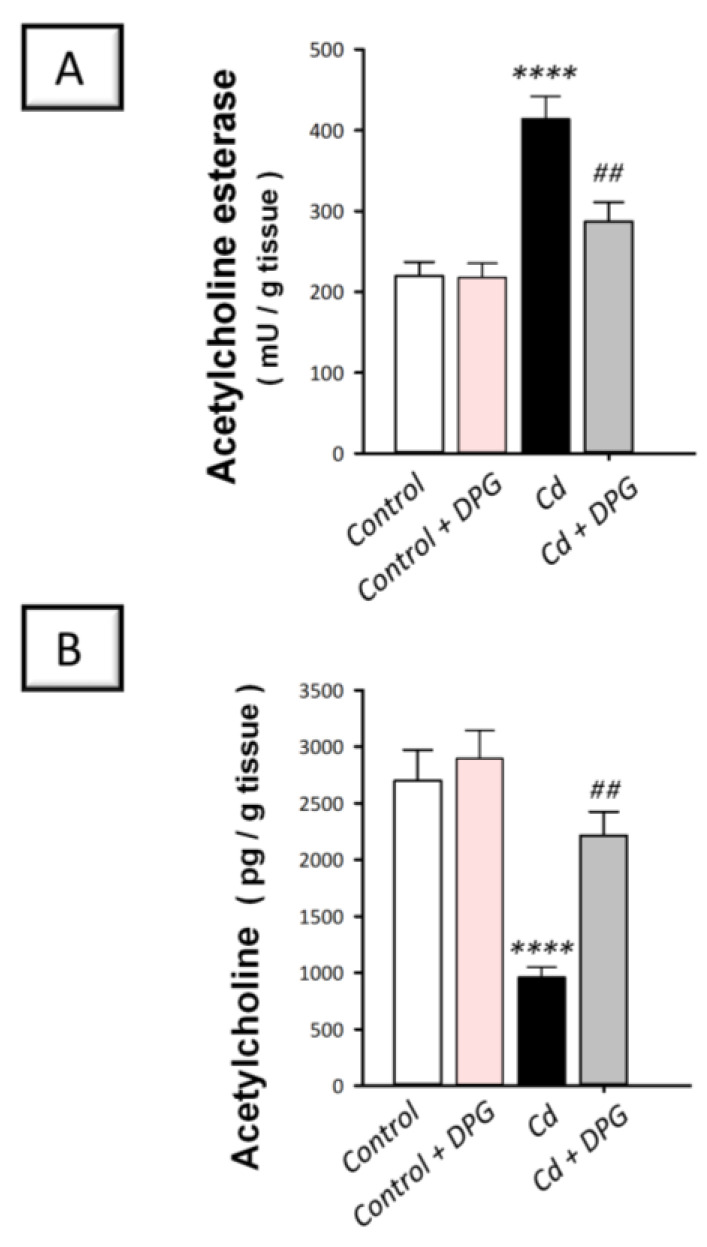
Dapagliflozin decreased the hippocampal activity of acetylcholine esterase while enhancing the levels of acetylcholine in cadmium-intoxicated rats. (**A**) Acetylcholine esterase activity. (**B**) The acetylcholine levels. The mean ± standard error (SE) was presented in the graph (*n* = 6). Statistical comparisons versus the control group were demonstrated by **** *p* < 0.0001. Statistical comparisons versus the Cd group were demonstrated by ^##^ *p* < 0.01 (Bonferroni’s test and one-way ANOVA). Cd, cadmium chloride (5 mg/kg/day, via gavage for 2 months); DPG, dapagliflozin (1 mg/kg/day, by gavage for 2 months).

To investigate cognitive decline and Alzheimer-like neuropathology, we investigated the hippocampal activity of acetylcholine esterase and its neurotransmitter substrate acetylcholine in cadmium-intoxicated rats. Indeed, the cognitive impairment is principally prompted by defective cholinergic transmission in the hippocampus [23]. In Figure 5A,B, a statistically significant (*p* < 0.0001) elevation was observed in hippocampal acetylcholine esterase activity (by 88.4%) whereas a statistically significant (*p* < 0.0001) decline was seen in acetylcholine levels (by 64.4%) in rats intoxicated with cadmium (acetylcholine esterase activity: F (3, 20) = 17.46, *p* < 0.0001; acetylcholine: F (3, 20) = 15.94, *p* < 0.0001). These changes were reversed by DPG as shown by the statistically significant (*p* < 0.01) reduction in acetylcholine esterase activity by 30.6% together with the statistically significantly (*p* < 0.01) increased acetylcholine levels by 130.4%. These data suggest that increasing hippocampal acetylcholine while lowering acetylcholine esterase activity contributes, at least in part, to the amelioration of cadmium-evoked cognitive impairment in rats.

### 3.6. Dapagliflozin Stimulated Hippocampal Impaired Autophagy Events and Stimulated AMPK/mTOR Pathway in Cadmium-Intoxicated Rats

**Figure 6:**
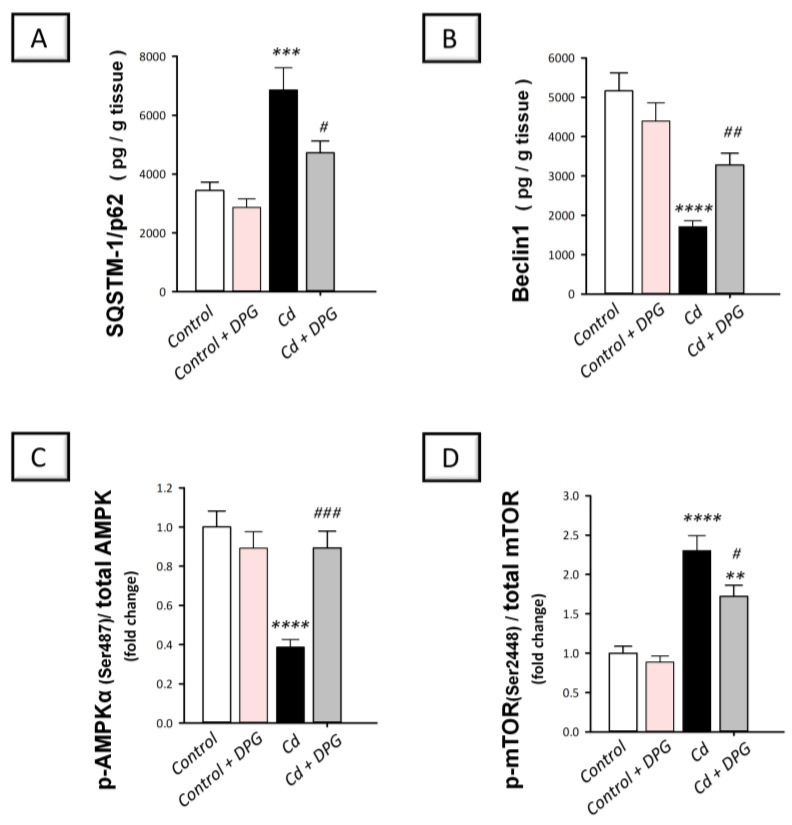
Dapagliflozin rescued impaired hippocampal autophagy events in cadmium-intoxicated rats. (**A**) The protein expression of sequestosome-1/protein 62 (SQSTM-1/p62) was reduced by dapagliflozin. (**B**) The protein expression of Beclin 1 was enhanced by dapagliflozin. Moreover, dapagliflozin elevated the phosphorylated adenosine-monophosphate-activated protein kinase (p-AMPK)/total AMPK (**C**) and diminished the phosphorylated mechanistic target of rapamycin (p-mTOR)/total mTOR in the hippocampus tissue (**D**), indicating that AMPK/mTOR pathway was stimulated. The mean ± standard error (SE) was presented in the graph (*n* = 6). Statistical comparisons versus the control group were demonstrated by **** *p* < 0.0001, *** *p* < 0.001, or ** *p* < 0.01. Statistical comparisons versus the Cd group were demonstrated by ^###^ *p* < 0.001, ^##^ *p* < 0.01, or ^#^ *p* < 0.05 (Bonferroni’s test and one-way ANOVA). Cd, cadmium chloride (5 mg/kg/day, via gavage for 2 months); DPG, dapagliflozin (1 mg/kg/day, by gavage for 2 months).

Contradictory findings have been reported on cadmium’s impact on autophagy in neuronal cells in vitro [9,10,12]. Moreover, few reports have explored the effect of cadmium on hippocampus autophagy in vivo. As part of our investigation, we examined SQSTM-1/p62 expression, a marker of impeded autophagy indicating a dysfunction of autophagosome degradation [3], together with Beclin 1 autophagy marker. In the same regard, the pro-autophagic AMPK/mTOR pathway was examined in the hippocampi. In Figure 6A,B, a statistically significant (*p* < 0.001) increase was observed in SQSTM-1/p62 by 99.4% whereas a statistically significant (*p* < 0.0001) lowering was seen in Beclin 1 by 66.8.% in rats intoxicated with cadmium (SQSTM-1/p62: F (3, 20) = 14.25, *p* < 0.0001; Beclin 1: F (3, 20) = 15.44, *p* < 0.0001). This was associated with hippocampal AMPK/mTOR pathway suppression in rats intoxicated with cadmium (Figure 6C,D). In this context, a statistically significant (*p* < 0.0001) decrease was observed in p-AMPK/total AMPK by 61.3% whereas a statistically significant (*p* < 0.0001) elevation was shown in p-mTOR/total mTOR by 130.4% (p-AMPK/total AMPK: F (3, 20) = 13.37, *p* < 0.0001; p-mTOR/total mTOR: F (3, 20) = 25.03, *p* < 0.0001). In response to DPG administration, SQSTM-1/p62 protein expression demonstrated a statistically significant (*p* < 0.05) decrease and Beclin 1 levels showed a statistically significant (*p* < 0.01) elevation by 31.2% and 91.8%, respectively. These events proved that the hippocampal defective autophagy was rescued by DPG. These favorable events were associated with DPG-evoked stimulation of AMPK/mTOR pathway, as evidenced by a statistically significant (*p* < 0.001) elevation in p-AMPK/total AMPK and a statistically significant (*p* < 0.05) decline in p-mTOR/total mTOR by 130.9% and 25.3%, respectively. Overall, DPG-triggered stimulation of autophagy and associated AMPK/mTOR pathway in the hippocampus contributes, at least in part, to the amelioration of cadmium-evoked cognitive impairment in rats.

### 3.7. Dapagliflozin Reversed the Apoptotic Events in Cadmium-Intoxicated Rats

**Figure 7:**
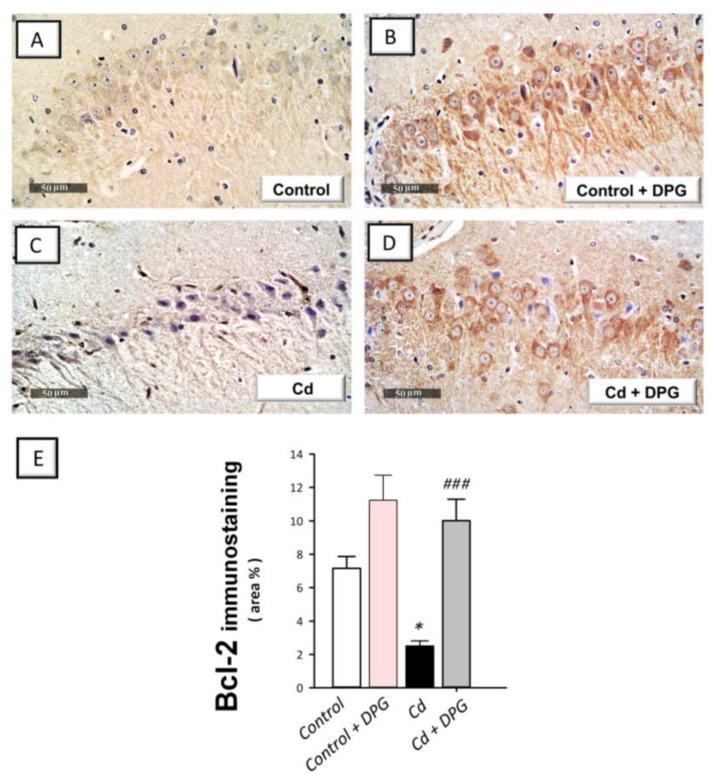
Dapagliflozin upregulated hippocampal B-cell lymphoma-2 protein (Bcl-2) immunostaining in cadmium-intoxicated rats. (**A**–**D**) Immunohistochemical staining of Bcl-2 in hippocampal CA3 region (revealed as brown staining; scale bar: 50 µm). (**E**) Bcl-2 quantitation is depicted in the graph (staining area % relative to the entire area of the microscopic field; in each section, 6 non-overlapping microscopic fields were inspected). The mean ± standard error (SE) was presented in the graph (*n* = 6). Statistical comparisons versus the control group were demonstrated by * *p* < 0.05. Statistical comparisons versus the Cd group were demonstrated by ^###^ *p* < 0.001 (Bonferroni’s test and one-way ANOVA). Cd, cadmium chloride (5 mg/kg/day, via gavage for 2 months); DPG, dapagliflozin (1 mg/kg/day, by gavage for 2 months).

**Figure 8:**
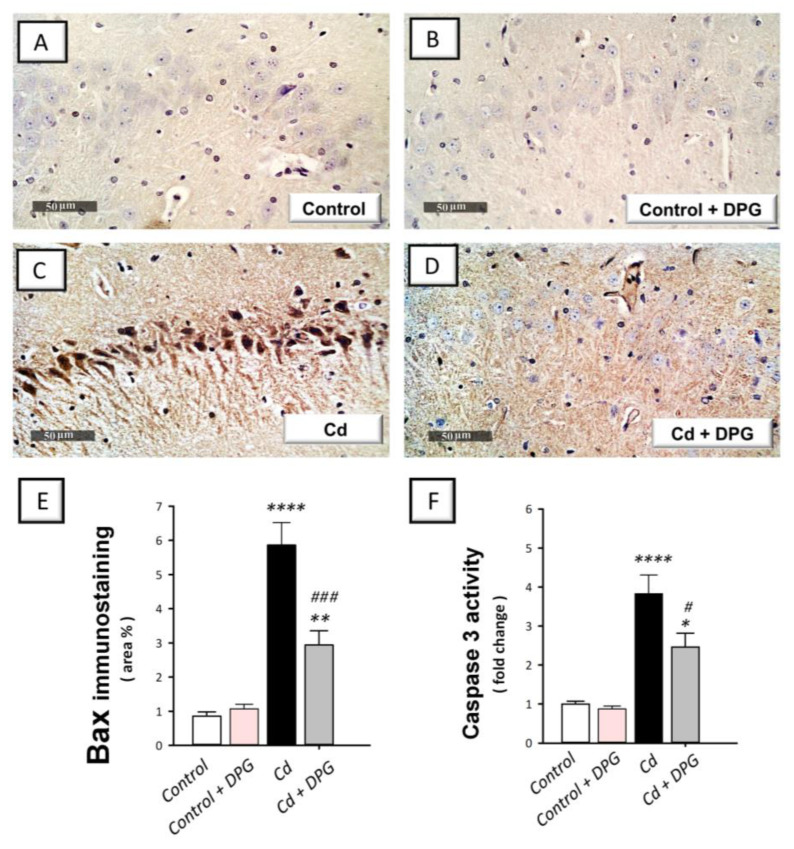
Dapagliflozin dampened hippocampal pro-apoptotic markers in cadmium-intoxicated rats. (**A**–**D**) Bcl-2 associated x protein (Bax) immunohistochemical staining in hippocampal CA3 region (revealed as brown staining; scale bar: 50 µm). (**E**) Bax quantitation is depicted in the graph (staining area % relative to the entire area of the microscopic field; in each section, 6 non-overlapping microscopic fields were inspected). (**F**) The activity of hippocampal caspase 3. The mean ± standard error (SE) was presented in the graph (*n* = 6). Statistical comparisons versus the control group were demonstrated by **** *p* < 0.0001, ** *p* < 0.01, or * *p* < 0.05. Statistical comparisons versus the Cd group were demonstrated by ^###^ *p* < 0.001, or ^#^ *p* < 0.05 (Bonferroni’s test and one-way ANOVA). Cd, cadmium chloride (5 mg/kg/day, via gavage for 2 months); DPG, dapagliflozin (1 mg/kg/day, by gavage for 2 months).

Defective neuronal autophagy has been linked to the induction of pro-apoptotic responses as demonstrated in neuronal cells in vitro, according to a large body of evidence [12]. Meanwhile, there is a significant correlation between hippocampal apoptosis and behavioral impairment in rodent models of cadmium neurotoxicity [5,6]. In rats intoxicated with cadmium, Bcl-2 protein expression (Figure 7E) showed a statistically significant (*p* < 0.05) decrease by 64.9% (F (3, 20) = 13.39, *p* < 0.0001) whereas Bax protein expression (Figure 8E) demonstrated a statistically significant (*p* < 0.0001) elevation by 589% (F (3, 20) = 33.92, *p* < 0.0001). In the same regard, cadmium induced a statistically significant (*p* < 0.0001) elevation in hippocampal caspase 3 activity (Figure 8F) by 283% (F (3, 20) = 21.03, *p* < 0.0001). These pro-apoptotic events in cadmium-intoxicated rats were reversed by DPG as evidenced by the statistically significantly (*p* < 0.001) upregulated protein expression of Bcl-2 and the statistically significantly (*p* < 0.001) downregulated expression of Bax by 298% and 49.9%, respectively. In tandem, DPG statistically significantly (*p* < 0.05) reduced caspase 3 activity by 35.7% in the hippocampus tissue of cadmium-intoxicated rats. Overall, DPG-triggered dampening of hippocampal pro-apoptotic response contributes, at least in part, to the amelioration of cadmium-induced cognitive impairment in rats.

### 3.8. Dapagliflozin Reversed Hippocampal Pro-Oxidant Events in Cadmium-Intoxicated Rats

**Figure 9:**
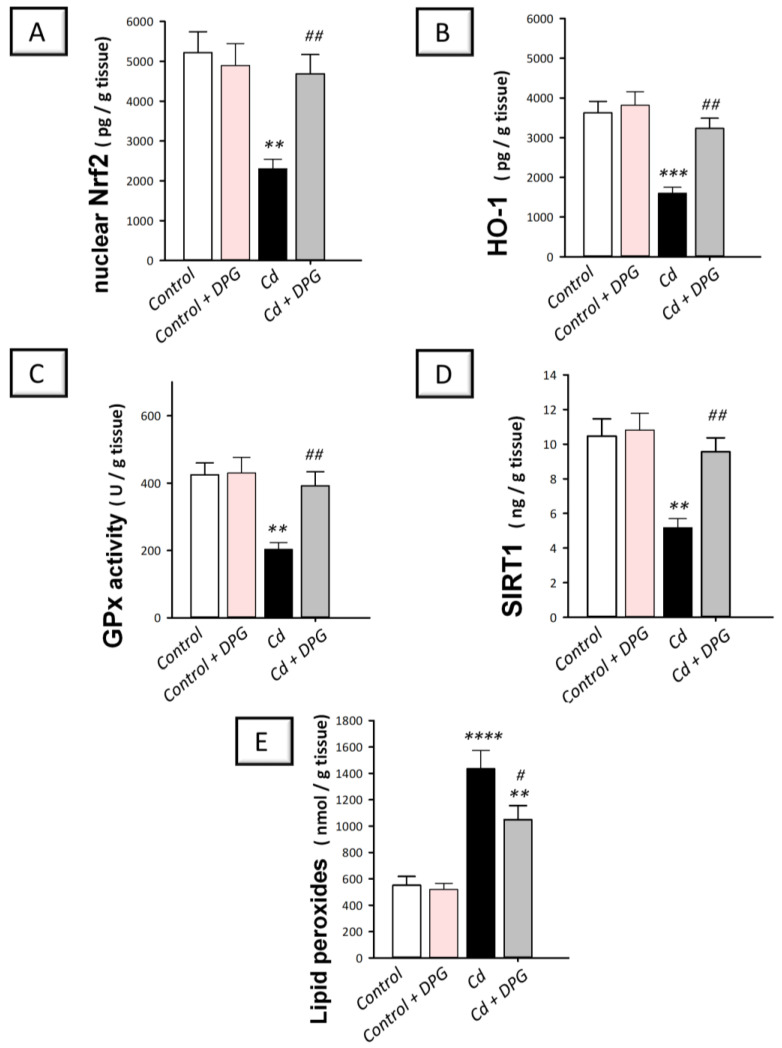
Dapagliflozin stimulated hippocampal antioxidant response in cadmium-intoxicated rats. Dapagliflozin stimulated Nrf2/HO-1 pathway by augmenting the hippocampal levels of nuclear factor erythroid-2-related factor-2 (Nrf2; (**A**)), heme oxygenase-1 (HO-1; (**B**)), and glutathione peroxidase (GPx; (**C**)), together with the upstream cytoprotective signal silent-information-regulated transcription factor 1 (SIRT1; (**D**)). Meanwhile, dapagliflozin lowered the hippocampal lipid peroxide levels (**E**) in cadmium-intoxicated rats. The mean ± standard error (SE) was presented in the graph (*n* = 6). Statistical comparisons versus the control group were demonstrated by **** *p* < 0.0001, *** *p* < 0.001, or ** *p* < 0.01. Statistical comparisons versus the Cd group were demonstrated by ^##^ *p* < 0.01, or ^#^ *p* < 0.05 (Bonferroni’s test and one-way ANOVA). Cd, cadmium chloride (5 mg/kg/day, via gavage for 2 months); DPG, dapagliflozin (1 mg/kg/day, by gavage for 2 months).

The exaggerated oxidative events in the hippocampi of rodents are closely associated with neuronal degeneration and memory disruption [4,5]. Therefore, we measured the hippocampal levels of lipid peroxide alongside the antioxidant SIRT1/Nrf2/HO-1 axis. In rats intoxicated with cadmium (Figure 9A–C), hippocampal protein levels of nuclear Nrf2 (*p* < 0.01) and its downstream signals, such as HO-1 (*p* < 0.001), and GPx (*p* < 0.01) demonstrated a statistically significant decrease by 55.9%, 55%, and 52.2%, respectively (Nrf2: F (3, 20) = 8.172, *p* = 0.0010; HO-1: F (3, 20) = 14.05, *p* < 0.0001; GPx: F (3, 20) = 8.201, *p* = 0.0009). In the same context, Figure 9D,E revealed that cadmium induced a statistically significant (*p* < 0.01) decline in SIRT1 protein expression by 50.6% whereas the lipid peroxides showed a statistically significant (*p* < 0.0001) increase by 160.9% (SIRT1: F (3, 20) = 5.896, *p* = 0.0047; lipid peroxides: F (3, 20) = 20.64, *p* < 0.0001). These pro-oxidant events in cadmium-intoxicated rats were reversed by DPG as evidenced by the statistically significantly upregulated protein expression of Nrf2 (*p* < 0.01), HO-1 (*p* < 0.01), and GPx (*p* < 0.01), by 103.6%, 102.1%, and 92.8%, respectively. In tandem, DPG statistically significantly (*p* < 0.01) upregulated SIRT1 protein expression in the hippocampus tissue by 84.7% and statistically significantly (*p* < 0.05) diminished the lipid peroxides by 27%. Overall, DPG-triggered dampening of the pro-oxidant milieu contributes, at least in part, to the amelioration of cadmium-evoked cognitive impairment in rats.

## 4. Discussion

**Figure 10:**
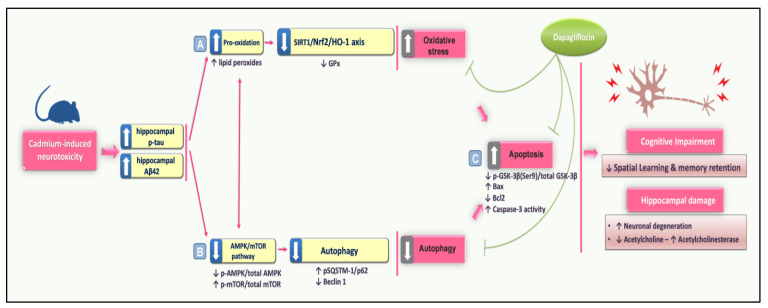
An outline that depicts the molecular events that mediated dapagliflozin’s neuroprotective impact against cadmium-induced cognitive dysfunction in rats. Dapagliflozin improved cadmium-induced behavioral outcomes and histopathological findings by augmenting cholinergic neurotransmission, curbing neurotoxic signals, e.g., hippocampal p-tau and Aβ42, and turning off GSK-3β activation. These beneficial effects were interceded by (A) Augmentation of SIRT1/Nrf2/HO-1 antioxidant axis and curbing hippocampal oxidation; (B) Stimulation of hippocampal AMPK/mTOR pathway and the linked autophagy stimulation; (C) Inhibition of the pro-apoptotic events in the hippocampus. Herein, solid arrows are indicative of activation whereas blunt arrows are indicative of inhibition.

The present study provides in vivo evidence for dapagliflozin (DPG)’s neuroprotection in rats against cadmium-evoked cognitive impairment and the linked neuropathology. Overall, DPG diminished hippocampal p-tau and Aβ_42_ and curtailed the noxious GSK-3β activation while augmenting the neuronal acetylcholine. These favorable actions were mediated by curbing hippocampal oxidative changes with SIRT1/Nrf2/HO-1 axis stimulation. In addition, DPG counteracted the apoptotic machinery and stimulated AMPK/mTOR-directed autophagy (Figure 10).

The cognitive impairments linked to recurrent cadmium exposure have been well documented [2]. Cadmium levels were shown to be greater in postmortem brain specimens of Alzheimer’s patients than in healthy controls, according to human epidemiological investigations [1]. As a result of crossing the BBB, cadmium has been reported to accumulate in several brain regions including the hippocampus which is principally responsible for learning and memory functions [4,5]. Consistently, the present work revealed cognitive deficits accompanied by a spike of the neurotoxic signals p-tau and Aβ_42_ alongside activation of their upstream effector GSK-3β. These events have been linked to neuronal death and synaptic disruption, leading to memory loss [3,5]. The multi-pronged GSK-3β is also reported to abrogate acetylcholine neurotransmitter; an event that has been previously characterized in cadmium-induced neurotoxicity [1,3].

Growing evidence revealed that therapeutic agents that can curtail p-tau and Aβ42 noxious signals and augment cholinergic transmission in the hippocampus of rodents can improve cognitive decline and memory disruption [23,32]. Consistently, the present study revealed that DPG rescued the cognitive decline in Cd-intoxicated rats by diminishing p-tau and Aβ42 alongside enhancing acetylcholine and dampening acetylcholine esterase activity. In the context of neurodegeneration, DPG demonstrated remarkable neuroprotection against rotenone-induced Parkinson’s disease [16] and 3-nitropropionic acid-induced Huntington-like manifestations [17]. In diabetic mice, DPG has been reported to enhance recognition memory, resulting in neuroprotection [46].

The process of autophagy is crucial for maintaining synaptic plasticity and neuronal homeostasis. These events promote memory formation and learning [10]. Hence, in vitro models of neurodegeneration demonstrated impaired autophagy flux in Neuro-2a cells and pheochromocytoma (PC12) [10,12]. In tandem, preclinical models and human AD postmortem brain specimens affirmed autophagy dysfunction [47]. In perspective, impaired elimination of autophagic vacuoles results in the accumulation of amyloid deposits [48]. In agreement with these data, the present study characterized hippocampal depletion of Beclin 1—a marker for autophagosome production at the sequestration phase [47], and accumulation of SQSTM-1/p62—a dysfunctional autophagy marker [3]. Together, these responses point to impaired autophagy events. Virtually, autophagy flux represents the sequence of processes which include autophagosome formation, transport of damaged mitochondria/protein aggregates cargo to the lysosome, and ultimately lysosomal enzyme degradation of this cargo [47]. Of note, the present finding of defective hippocampal autophagy may conflict with the previously reported autophagy stimulation in an in vivo model of cadmium-evoked neurotoxicity [9]. The disparity could be explained by the differences in cadmium exposure time, species of animal (rat vs. mouse), and extent of hippocampal/cognitive deficit [6,9].

Autophagy stimulation has been characterized as a critical clearance mechanism for neuronal p-tau/neurofibrillary tangles [49] and Aβ aggregates [9]. In rodent models of dementia, interventions that can stimulate autophagy, such as rapamycin have been shown to improve the behavioral phenotype and associated tau neuropathology [3,9]. Consistently, DPG stimulated hippocampal autophagy response, as revealed by Beclin 1 augmentation and SQSTM-1/p621 clearance, according to the current findings. Virtually, evidence exists that autophagy activation is one mechanism underlying the protective effects of DPG against 3-nitropropionic acid-induced Huntington-like disease [17]. Likewise, it has been demonstrated that DPG possesses pro-autophagic properties that mediate the mitigation of renal injury [50], testicular injury [33], inflammatory bowel disease [34], and hepatic steatosis [35]. In previous animal models, activation of the AMPK/mTOR pro-autophagic pathway interceded autophagy stimulation [34,35,50]. This notion is advocated by the present findings of hippocampal AMPK/mTOR pathway stimulation in response to DPG administration. Interestingly, the elimination of amyloid aggregates and p-tau in response to the activation of AMPK/mTOR pathway has been reported to improve cognitive deficits and neuropathology of Alzheimer’s disease [3]. Indeed, several AMPK activators such as quercetin and resveratrol curbed neurotoxicity in rodents by enhancing neuronal Aβ elimination [3,4].

Cadmium metal exposure causes neuronal apoptosis, primarily through the mitochondrial pathway, as the mechanism of cell death [5,6]. In response to cadmium, disruption of neuronal autophagy and accumulation of autophagosomes have been shown to instigate neuronal apoptotic death [36]. The later event is characterized by Bax overexpression and Bcl-2 dampened expression [5,6,8]. In accordance, the present study displayed that cadmium triggered several apoptotic events and stimulated the pro-apoptotic kinase GSK-3β. In response to DPG, these pro-apoptotic events were reversed, resulting in the abrogation of cadmium-triggered hippocampal neuronal loss and rescue of memory disruption. Indeed, marked anti-apoptotic features of DPG have been demonstrated in rotenone-induced Parkinson’s disease [16] and 3-nitropropionic acid-induced Huntington-like pathology [17]. In the same direction, the present findings further revealed that DPG inhibited GSK-3β, thereby promoting suppression of hippocampal neuronal apoptosis. In fact, GSK-3β inactivation has been reported to curb apoptosis by augmenting the anti-apoptotic signals, such as Bcl-2. In the same regard, DPG’s observed and published [34,35,50] stimulation of autophagy response supports neuronal pro-survival signals via elimination of p-tau/neurofibrillary tangles [49], aberrant Aβ aggregates and damaged mitochondria [9].

Cadmium-evoked neurotoxicity is associated with excessive pro-oxidant events as demonstrated in several in vitro experiments and hippocampal injury/cognitive deficit models in rodents [4,5]. Indeed, ample evidence suggests that free radical injury is a central cause of neuronal death linked to AD cognitive decline. This is supported by numerous studies in rodent models that have characterized several oxidative markers in AD [51]. The clinical data has also shown increased levels of oxidized nucleosides in the cerebrospinal fluid of AD patients including 8-oxo-7,8-dihydro-guanosine (8oxoGuo) and 8-oxo-7,8-dihydro-2′-deoxyguanosine (8oxodGuo) [52]. Interestingly, in vitro and in vivo experiments have shown that drugs with antioxidant properties can dampen β-amyloid fibril formation. In tandem, several antioxidant compounds have been shown to reduce the amyloid plaque burden when studied in transgenic mice [51,53]. In non-transgenic rodent models, agents with antioxidant potential have been proven to rescue the cognitive deficit phenotype and hippocampal neurodegeneration by augmenting neuronal GPx and combating hippocampal pro-oxidant signals including NADPH oxidase and inducible nitric oxide synthase [54]. Consistently, the present findings demonstrated excessive hippocampal pro-oxidant events and dampened SIRT1/Nrf2/HO-1 axis. These pro-oxidant aberrations were reversed by DPG. Indeed, DPG has been characterized to elicit remarkable antioxidant effects in rotenone-induced PD [34], obesity-induced cognitive decline [20], and myocardial infarction via suppression of ROS [14]. These antioxidant features can be attributed to the observed DPG-evoked GSK-3β inactivation which has been reported to dampen neuronal pro-oxidant events and associated neurodegeneration in several neuronal injury models [34,55,56]. Moreover, the observed DPG-induced SIRT1 upregulation has been characterized to attenuate cognitive decline via curtailing tau phosphorylation and amyloid plaque accumulation [7,57]. At the molecular level, SIRT1 has conferred neuroprotection in senile mice model via AMPK/mTOR pathway stimulation and the linked autophagy activation [53]. Moreover, SIRT1 can activate Nrf2 transcriptional ability and boost HO-1 and GPx antioxidants, leading to curtailing cadmium-induced neurotoxicity in cellular and experimental models [6,58]. There is also evidence that crosstalk exists between the cytoprotective Nrf2 and autophagy, with Nrf2 increasing the transcription of several autophagy-related genes such as *LAMP2A* and *SQSTM-1/p62* [59].

## 5. Conclusions

The present findings show that dapagliflozin has a neuroprotective impact in rats against cadmium-induced cognitive deficits. Dapagliflozin decreased hippocampal p-tau and Aβ42 and curtailed the pro-apoptotic GSK-3 activation while enhancing neuronal acetylcholine. These favorable actions were driven by dapagliflozin’s pro-autophagic, anti-apoptotic, and antioxidant features, resulting in behavioral recovery. Therefore, dapagliflozin may attenuate cadmium-induced cognitive deficits and neurotoxicity. Yet, further investigations are suggested to delineate dapagliflozin’s exact molecular mechanisms.

## Figures and Tables

**Table 1:** Experimental protocol.

Group	*N*	Received
Control	10	Group I received normal saline by gavage. Furthermore, animals received 0.5% carboxymethyl cellulose (CMC) by gavage. There was a two-hour interval between the two doses and the treatment protocol lasted for 2 months.
Control + DPG	10	Group II received normal saline by gavage. Furthermore, animals received dapagliflozin (1 mg/kg/day) by gavage. There was a two-hour interval between the two doses and the treatment protocol lasted for 2 months.
Cd	10	Group III received cadmium chloride (5 mg/kg/day) by gavage. Furthermore, animals received CMC by gavage. There was a two-hour interval between the two doses and the treatment protocol lasted for 2 months. The experimental regimen coincides with previously reported studies [22,23,24,25,26].
Cd + DPG	10	Group IV received cadmium chloride (5 mg/kg/day) by gavage. Furthermore, animals received dapagliflozin (1 mg/kg/day) by gavage. There was a two-hour interval between the two doses and the treatment protocol lasted for 2 months. Prior studies were used to determine the dose of dapagliflozin as an effective dose for the attenuation of the behavioral deficits in Huntington-like manifestations [17], chronic stress-triggered depression-like behavior [19], rotenone-induced Parkinson’s disease [16], PTZ-induced epilepsy [27], and high-fat diet-fed rats [18]. Furthermore, dapagliflozin was administered in rats at a dose that is similar to the dosage regimen typically used in humans, according to the human equivalent dose (HED) calculation technique [28].

## Data Availability

Data are contained within the article.
